# Supplementary material for: What happens to patient experience when you want to see a doctor and you get to speak to a nurse? Observational study using data from the English General Practice Patient Survey
Source: BMJ Open. 2018 Feb 3;8(2):e018690. doi: 10.1136/bmjopen-2017-018690 (PMC5829817; doi:10.1136/bmjopen-2017-018690)
Supplement: Supplementary file 1 [file bmjopen-2017-018690supp001.pdf]

Appendix Table 1. Five category summary of responses to the question “What did you want to do?” in the GP Patient Survey

	Response options to question: Last time you wanted to see or speak to a GP or nurse from your GP surgery: What did you want to do?*					
	See a GP at the surgery	See a nurse at the surgery	Speak to a GP on the phone	Speak to a nurse on the phone	Have someone visit me at my home	I didn’t mind / wasn’t sure what I wanted
Wanted to see or speak to a GP (or both)	X					
			X			
	X		X			
Wanted to see or speak to a Nurse (or both)		X				
				X		
		X		X		
Wanted a home visit					X	
Wasn’t sure or didn’t mind						X
Wanted more than one of the previous four categories	Any Other combination					

*Response options available to primary care patients completing the 2013/14 General Practice Patient Survey (http://www.gp-patient.co.uk).
