# Supplementary material for: What happens to patient experience when you want to see a doctor and you get to speak to a nurse? Observational study using data from the English General Practice Patient Survey
Source: BMJ Open. 2018 Feb 3;8(2):e018690. doi: 10.1136/bmjopen-2017-018690 (PMC5829817; doi:10.1136/bmjopen-2017-018690)
Supplement: Supplementary file 2 [file bmjopen-2017-018690supp002.pdf]

Appendix Table 2. Two category summary of responses to the question “What did you want to do?” in the GP Patient Survey

	Last time you wanted to see or speak to a GP or nurse from your GP surgery: What did you want to do?					
	See a GP at the surgery	See a nurse at the surgery	Speak to a GP on the phone	Speak to a nurse on the phone	Have someone visit me at my home	I didn't mind / wasn't sure what I wanted
Wanted to see or speak to a GP (or both)	**X**					
			**X**			
	**X**		**X**			
	**X**					**X***
			**X**			**X***
	**X**		**X**			**X***
Wanted to see or speak to a Nurse (or both)		**X**				
				**X**		
		**X**		**X**		
		**X**				**X***
				**X**		**X***
		**X**		**X**		**X***
Excluded	Any other combination					

*Where a patient indicated they didn't mind while only indicating an appointment with either a GP or nurse, but not both, we took this to indicate they did not mind how they interacted with the healthcare professional, but they had expressed a preference for a particular type of health professional
