# Supplementary material for: What happens to patient experience when you want to see a doctor and you get to speak to a nurse? Observational study using data from the English General Practice Patient Survey
Source: BMJ Open. 2018 Feb 3;8(2):e018690. doi: 10.1136/bmjopen-2017-018690 (PMC5829817; doi:10.1136/bmjopen-2017-018690)
Supplement: Author's manuscript [file bmjopen-2017-018690.draft_revisions.pdf]

BMJ Open

BMJ Open is committed to open peer review. As part of this commitment we make the peer review history of every article we publish publicly available.

When an article is published we post the peer reviewers' comments and the authors' responses online. We also post the versions of the paper that were used during peer review. These are the versions that the peer review comments apply to.

The versions of the paper that follow are the versions that were submitted during the peer review process. They are not the versions of record or the final published versions. They should not be cited or distributed as the published version of this manuscript.

BMJ Open is an open access journal and the full, final, typeset and author-corrected version of record of the manuscript is available on our site with no access controls, subscription charges or pay-per-view fees (http://bmjopen.bmj.com).

If you have any questions on BMJ Open's open peer review process please email editorial.bmjopen@bmj.com

What happens to patient experience when you want to see a doctor and you get to speak to a nurse? Observational study using data from the English General Practice Patient Survey

Journal:	*BMJ Open*
Manuscript ID	bmjopen-2017-018690
Article Type:	Research
Date Submitted by the Author:	31-Jul-2017
Complete List of Authors:	Paddison, Charlotte; Nuffield Trust, ; University of Cambridge, Institute of Public Health<br>Abel, Gary; University of Exeter, University of Exeter Medical School (Primary Care)<br>Burt, Jenni; University of Cambridge, Cambridge Centre for Health Services Research<br>Campbell, John; University of Exeter, Primary Care;<br>Elliot, Marc; RAND corporation,<br>Lattimer, Valerie; UEA, School of Nursing Sciences<br>Roland, Martin; Institute of Public Health, Institute of Public Health
**Primary Subject Heading**:	General practice / Family practice
Secondary Subject Heading:	Health services research, Health policy
Keywords:	Health services research, PRIMARY CARE, patient experience, Quality in health care < HEALTH SERVICES ADMINISTRATION & MANAGEMENT

For peer review only - http://bmjopen.bmj.com/site/about/guidelines.xhtml

Title:

What happens to patient experience when you want to see a doctor and you get to speak to a nurse? Observational study using data from the English General Practice Patient Survey

Authors:

Charlotte A. M. Paddison PhD [1], Gary A. Abel PhD [2], Jenni Burt PhD [3], John Campbell MD FRCGP [2], Marc N. Elliott PhD [4], Valerie Lattimer [5], Martin Roland DM FRCGP [3]

[1] Nuffield Trust, 59 New Cavendish Street, London, UK W1G 7LP

[2] University of Exeter Medical School, St Lukes Campus, Magdalen Road, Exeter EX1 2LU, UK

[3] Cambridge Centre for Health Services Research, Primary Care Unit, Institute of Public Health, Forvie Site, University of Cambridge School of Clinical Medicine, Box 113 Cambridge Biomedical Campus, Cambridge, CB2 0SR, UK

[4] RAND Corporation, 1776 Main Street, P.O. Box 2138, Santa Monica, CA 90407-2138

[5] University of East Anglia, School of Nursing Science, Norwich Research Park, Norwich, Norfolk UK NR4 7TJ

Corresponding author

Charlotte Paddison

Nuffield Trust, 59 New Cavendish Street, London, UK W1G 7LP

Phone: 0207 462 0548

Email: (academic) camp3@medschl.cam.ac.uk

For peer review only - http://bmjopen.bmj.com/site/about/guidelines.xhtml

Secondary email: (non-academic/employer) charlotte.paddison@nuffieldtrust.org.uk

Keywords

patient experience, health services research, primary care, health care quality

Word count (manuscript, excluding title page, references, figures): 2,847

For peer review only

Abstract

Objectives: To examine patient consultation preferences for seeing or speaking to a GP or nurse; to estimate associations between patient-reported experiences and the type of consultation patients actually received (phone or face-to-face, GP or nurse)

Design: Secondary analysis of data from the 2013/14 GP Patient Survey

Setting and participants: 870,085 patients from 8,005 English general practices

Outcomes: Patient ratings of communication and 'trust and confidence' with the clinician they saw.

Results: 77.7% of patients reported wanting to see or speak to a GP, while 14.5% reported asking to see or speak to a nurse the last time they tried to make an appointment (weighted percentages). Being unable to see or speak to the practitioner type of the patients' choice was associated with lower ratings of trust and confidence and patient-rated communication. Smaller differences were found if patients wanted a face-to-face consultation and received a phone consultation instead. The greatest difference was for patients who asked to see a GP and instead spoke to a nurse for whom the adjusted mean difference in confidence and trust compared to those who wanted to see a nurse and did see a nurse was -15.8 points (95% CI -17.6, -14.0) for confidence and trust in the practitioner and -10.5 points (95%CI -11.68, -9.27) for net communication score, both on a 0-100 scale.

Conclusions: Patients' evaluation of their care is worse if they do not receive the type of consultation they expect, especially if they prefer a doctor but are unable to see one. New models of care should consider the potential unintended consequences for patient experience of the widespread introduction of multi-disciplinary teams in general practice.

(Word count: 272 words)

Strengths and limitations of this study

- Large national survey with respondents including 870,085 patients from 8,005 English general practices
- Appointment choice (mode and type of practitioner), and patient-reported experience, all collected in a single survey.
- Thirty-four per cent response rate, though typical for similar national surveys.
- Patient-reported confidence and trust assessed using a single item within the GP Patient Survey.

For peer review only - http://bmjopen.bmj.com/site/about/guidelines.xhtml

Patient experience is a core dimension of health care quality[1] and measuring patient experience enables the views of service users to be incorporated into the evaluation and improvement of health services at local and national level.[,2,3] In the UK, 90% of all NHS patient contacts occur in primary care. Though primary care is highly valued by the UK public, the primary care workforce is under unprecedented strain[4,5] and clear policies for reforming primary care in England are now emerging.[6,7] These include the development of wider multi-disciplinary teams that make greater use of nurses, physicians' associates, pharmacists, and administrative support. It is anticipated that an expanded workforce will free up clinical time for doctors, enabling them to devote more time to the delivery of care for complex patients and spend less time on administration and minor health issues.

At present it is unclear what impact these proposed changes to the primary care workforce might have on patient choice and quality of care, including patient experience. Multi-disciplinary teams could increase patient choice by improving access to nurses and pharmacists, but their introduction could also have unintended adverse impacts, for example on patients who were no longer able to see the type of clinician of their choice.

Given the policy emphasis on promoting patient choice, evidence is needed on the acceptability to patients of seeing different practitioners, especially when their original intention had been to see a GP. Systematic reviews suggest that nurse-GP substitution in primary care can produce similar or better quality of care and high levels of patient satisfaction [8,9], but there is also evidence that some patients do not want nurses to substitute for GPs.[10] Relational continuity of care, together with perceived trust and confidence, have emerged as important factors in understanding why some patients may prefer to see a GP and, more specifically, a GP who knows them well.[11]

In this primary care-based study we examine patient consultation preferences and their association with patient-reported experiences, using data from the English General Practice Patient Survey (GPPS) to address the following questions:

1. To what extent do patients in primary care request an appointment with a doctor or a nurse, and how often is this request met?

2. When patients are unable to get an appointment of their choice (phone or face-to-face, GP or nurse), is this associated with poorer patient-reported communication or lower trust and confidence?

Methods

We analysed data from the 2013/14 General Practice Patient Survey (http://www.gp-patient.co.uk), which is sent as a mail questionnaire to 2.6 million patients in England each year[12]. We estimated preference for nurse and GP appointments from the question which asked respondents 'Last time you wanted to see or speak to a GP or nurse from your GP surgery: What did you want to do?' with response options coded into five categories (see Appendix Table 1):

- see or speak to a GP
- see or speak to a Nurse
- a home visit

For peer review only - http://bmjopen.bmj.com/site/about/guidelines.xhtml

- wasn't sure or didn't mind
- wanted more than one of the previous four categories

Practice population estimates were computed using survey weights that account for survey design and non-response.[13] Variation in preferences for GP and nurse appointments were explored using crude and adjusted logistic regression using a binary classification (wanting to see or speak to a nurse vs. wanting to see or speak to a GP as the outcome - see Appendix Table 2 for detail of how categories were constructed), excluding those indifferent as to whether they saw a doctor or a nurse. Fixed effects included in the models were patient reported age, gender, ethnicity, confidence in managing their own health, and presence of a long-term condition, as well as a measure of social deprivation based on the patient's postcode of residence (national quintiles as categories). General practice was included as a random effect (intercept). All analyses were restricted to patients with complete information on all variables included in the adjusted model.

Next we investigated whether people who did not get an appointment with the type of practitioner of their choice were more likely to report poorer patient experience. For simplicity this analysis was restricted to those who only endorsed one response option each for the question on what type of appointment they had requested and for the question on what they got. Sixteen categories, combinations of seeing or speaking to a health professional and whether the appointment was with a GP or a nurse, were created. The dataset was then split into those whose last appointment was with a GP and those whose last appointment was with a nurse, and these were modelled separately.

Two series of regression models were run. The first series of models used responses to the question 'Did you have confidence and trust in the GP (nurse) you saw or spoke to?' as the outcome. Response options were: 'yes, definitely'; 'yes, to some extent'; and 'no, not at all' which were scored as 100, 50 and 0 respectively. Crude differences between the categories were estimated for trust and confidence in GPs and, separately, for nurses, using linear regression. Mixed-effect linear regression was then used for an adjusted analysis including age, gender, ethnicity, confidence in managing their own health, the presence of a long standing heath condition, and deprivation. Practice was included as a random effect (intercept), and analyses were restricted to those who had complete information on all socio-demographic and health variables in this study.

A second series of models using the same approach (linear regressions to estimate crude differences between categories, and mixed-effect linear regression for analyses adjusting for health and demographic characteristics and additionally controlling for practice) were then run using composite patient-rated communication scores as the outcome. This composite score was the mean of all responses to five questions on patient reported experiences of communication for patients who answered at least three of these questions. Scores were computed separately for doctor communication (five items) and nurse communication (five items). The questions asked how good the GP or nurse was at 'giving you enough time', 'listening to you' or 'explaining tests and treatments', 'involving you in decisions about your care and 'treating you with care and concern'. As in our previous work, responses to these patient experience items were linearly rescaled to give a score from 0 to 100 as in previous work in order to facilitate comparison between items. [14,15]

For peer review only - http://bmjopen.bmj.com/site/about/guidelines.xhtml

Results

Among 870,085 primary care patients from 8005 practices in England, more than three quarters reported that they only asked to see or speak to a GP, whilst just under 15% only asked to see or speak to a nurse (Table 1) with the remainder wanting a home visit, expressing no preference or asking for multiple types of appointment. Comparing the type of appointment patients wanted and the type of appointment they actually received, we found that the great majority of people got what they wanted. As shown in Table 1, 96% of people who wanted to see or speak to a GP achieved this, compared to 92% of people who wanted to see or speak to a nurse. The number wanting a home visit who got one was lower at 80%.

<insert Table 1 approximately here>

There were 685,244 respondents who only wanted to see or speak to either a nurse or doctor who also had complete information for all covariates of interest. Of these 122,597 (weighted percentage 16.0%) wanted to see or speak to a nurse, with the remainder wanting to see or speak to a GP. Variation in this percentage is shown in Table 2 along with crude and adjusted odds ratios. Older patients were more likely to want to see or speak to a nurse than younger patients (e.g. 85 and over vs. 55 to 64 year olds adjusted OR=1.31 95% CI 1.26, 1.36). Ethnic minority group patients were much less likely to want to see or speak to a nurse (e.g. Asian vs. White patients adjusted OR=0.66 95% CI 0.64, 0.68). Similarly those who were not confident in managing their own health were less likely to want to see or speak to a nurse (e.g. Not at all confident vs. Very confident adjusted OR=0.58 95% CI 0.54, 0.62).

<insert Table 2 approximately here>

Being unable to get an appointment of the patients' choice was associated with differences in trust and confidence (Table 3) and communication (Table 4), among patients who reported seeing or speaking to a nurse or a GP in their last primary care consultation. The mean trust and confidence score for those who had asked to see a nurse and did so was 91.0 (on a scale of 0- 100). For all other combinations patient-rated trust and confidence scores were, on average, lower ($p<0.001$). Patient reported trust and confidence was lowest for those who wished to see a GP or wished to speak to a GP, and instead spoke to a nurse (adjusted mean difference compared to those who wanted to see a nurse and did see a nurse = -15.8 95% CI -17.6, -14.0, and -13.5 95% CI -15.9, -11.0, respectively), followed by those who wanted to see a GP but ended up speaking to, rather than seeing, a GP (-6.5, 95% CI -7.0, -5.9). Similar differences were found for patient reported communication scores, with the lowest scores for those who wanted to see a GP but spoke to a nurse (adjusted mean difference compared to those who wanted to see a nurse and did see a nurse = -10.5 95%CI -11.7, -9.2).

For peer review only - http://bmjopen.bmj.com/site/about/guidelines.xhtml

<insert Table 3 approximately here>

<insert Table 4 approximately here>

Discussion

In a study of 870,085 respondents from general practices in England, we show that the majority of patients in primary care are able to see or speak to the health professional type (GP vs nurse) of their choice. However a substantive minority - between four per cent and eight per cent - are not. We found evidence that patient consultation preferences vary across patient groups. Patients with low confidence in managing their own health, younger patients and ethnic minority groups were more likely to want to see a GP.

When patients were unable to obtain the type of appointment of their choice, this was associated with lower confidence and trust in the health professional they saw, and poorer patient-rated communication. This difference was particularly large among patients who wished to see a GP and instead spoke to a nurse. Patients whose appointments were with GPs reported similar experiences regardless of their initial preference for a nurse versus a doctor. Patient experience scores were also lower when the patient had asked to see a health professional (either a GP or a nurse), and ended up having a telephone call.

Our study, using data from a large national survey of patient experience in primary care, builds on what is known from previous research examining the acceptability to patients' of nurse-GP substitution[8,9] and extends on smaller qualitative studies of patients' preferences for GP versus nurse-led consultations[10,11]. In particular, this study highlights the possible unintended impacts of nurse-GP substitution on patients' confidence and trust, and the quality of communication in primary care. The response rate (34%) in our study is comparable with other major national surveys, and a previous assessment found no association between practice response rates and scores[16] Despite its large overall sample, one limitation of our study is the smaller size of some individual patient subgroups giving rise to larger uncertainty. Another limitation is that we assessed patient-reported confidence and trust using the single item that represents this concept in the GP Patient Survey, and future research might consider including additional items to provide a comprehensive and multidimensional measure of these constructs.

Changing nature of primary care and implications for patient confidence and trust

The role of nurses in primary care has changed considerably in the last 30 years, with the advent of advanced nurse practitioners, independent nurse prescriber roles, and the existence of some primary care practices that are led by nurses. Some patients appear uncertain about what to expect in nurse-led primary care consultations.[9,11] In our study we found that patients who got to speak to a nurse when they wished to see a GP, reported lowest confidence and trust in their eventual consultation. The magnitude of this effect represents a large difference of 15 points on a scale of 0 – 100, controlling for socio-demographic characteristics and health status and combines not seeing or speaking to the type of practitioner of their choice (i.e. nurse rather than doctor) and receiving a

For peer review only - http://bmjopen.bmj.com/site/about/guidelines.xhtml

telephone call when they had asked for a face to face consultation. There are several possible explanations for these findings. Patients may feel that they are being inappropriately denied access to GPs, resulting in frustration that could affect both trust and the quality of interpersonal communication (*gatekeeper hypothesis*). Lack of trust and confidence may also arise because some patients perceive that nurses are subordinate to GPs in terms of skills and knowledge (*patient confidence hypothesis*). Our findings are in line with the results from a pragmatic controlled trial of telephone triage among patients seeking a same-day GP appointment which showed that nurse-led triage was associated with somewhat worse patient-reported experience and lower overall patient satisfaction in comparison with usual care.[17]

Implications for health policy, clinical practice and primary care research

A strong vision for reforming primary care in England is now emerging.[7] This includes expanded roles for pharmacists, physician associates, and a £15 million investment in nursing capacity within General Practice. When new ways of working are introduced, primary care practices need to communicate these to patients who may know less about the care offered by health professionals other than GPs. Building patient confidence and educating patients about the skills of team members should form part of a broader effort to involve patients in the design and development of new services. New models of care should consider the possible intended and unintended consequences for patient experience of multi-disciplinary teams, particularly given the policy emphasis on enabling patient choice. Monitoring should include any potential adverse effects on patient confidence and trust in health professionals. Further work is needed to ascertain the impacts of proposed workforce changes on different patient groups. While proposals for nurses to help meet gaps in the supply of primary care providers have met with wide interest[18], there are important remaining uncertainties about the impact of this on patients' experiences and on the maintenance of confidence and trust in primary care in the NHS.

For peer review only

For peer review only - http://bmjopen.bmj.com/site/about/guidelines.xhtml

Last time you wanted to see or speak to a GP or nurse from your GP surgery: What did you want to do?	N (Weighted %)*	What type of appointment did you get?** To see and/or speak to a GP	To see and/or speak to a Nurse	A home visit	One or more of the above	Total
See and/or speak to a GP	653,526 (77.7%)	95.9%	2.9%	0.1%	1.1%	100.0%
See and/or speak to a Nurse	139,300 (14.5%)	6.3%	92.2%	0.1%	1.4%	100.0%
A home visit	12,873 (1.2%)	15.7%	2.2%	79.6%	2.5%	100.0%
Didn't mind/wasn't sure	15,404 (2.4%)	67.1%	29.1%	1.1%	2.7%	100.0%
One or more of the above	48,982 (4.3%)	29.7%	8.6%	0.7%	61.1%	100.0%
Total	870,085 (100.0%)	77.6%	17.5%	1.1%	3.8%	100.0%

*Weighted percentages are calculated using survey design and non-response weights (by age, gender, geographical location and GP practice, full details Technical Annex GP Patient Survey[13])

**Of those who answered the question 'Last time you wanted to see or speak to a GP or nurse from your GP surgery: What did you want to do?'(N = 870,085) only 754,551 also answered the question 'What type of appointment did you get?')

Table 1. Descriptive statistics showing what patients wanted to do last time they contacted their GP surgery, and weighted percentages showing the association between the type of appointment patients wanted and the appointment they got.

For peer review only - http://bmjopen.bmj.com/site/about/guidelines.xhtml

		Wanted GP appointment N (%)†‡	Wanted nurse appointment N (%)†	Crude OR Wanted GP appointment (95% CI)*	Adjusted OR Wanted GP appointment (95% CI)**
Age	18 to 24	23978(86.5%)	3953(13.5%)	1.34 (1.29, 1.39)	1.29 (1.24, 1.34)
	25 to 34	55900(86.1%)	9828(13.9%)	1.26 (1.22, 1.29)	1.20 (1.17, 1.23)
	35 to 44	78731(87.0%)	12575(13.0%)	1.38 (1.35, 1.41)	1.32 (1.29, 1.35)
	45 to 54	106693(85.2%)	19007(14.8%)	1.24 (1.21, 1.27)	1.22 (1.20, 1.24)
	55 to 64	117426(82.3%)	25927(17.7%)	Reference	Reference
	65 to 74	107427(77.9%)	31381(22.1%)	0.76 (0.74, 0.77)	0.77 (0.76, 0.79)
	75 to 84	57635(79.0%)	15802(21.0%)	0.81 (0.79, 0.82)	0.80 (0.79, 0.82)
	85 or over	14857(78.6%)	4124(21.4%)	0.80 (0.77, 0.83)	0.76 (0.74, 0.79)
Gender	Male	246540(85.2%)	53712(14.8%)	Reference	Reference
	Female	316107(82.8%)	68885(17.2%)	1.00 (0.99, 1.01)	0.99 (0.97, 1.00)
Ethnicity	White	495207(83.4%)	113381(16.6%)	Reference	Reference
	Mixed	4432(87.0%)	686(13.0%)	1.48 (1.36, 1.60)	1.23 (1.14, 1.33)
	Asian	33748(89.1%)	4303(10.9%)	1.80 (1.74, 1.85)	1.51 (1.47, 1.56)
	Black	14442(87.8%)	2151(12.2%)	1.54 (1.47, 1.61)	1.30 (1.25, 1.36)
	Other	14818(88.4%)	2076(11.6%)	1.63 (1.56, 1.71)	1.41 (1.35, 1.47)
IMD group	1 – least deprived	111565(83.3%)	25422(16.7%)	Reference	Reference
	2	116118(83.1%)	26848(16.9%)	0.99 (0.97, 1.00)	0.98 (0.97, 1.00)
	3	115657(83.5%)	26334(16.5%)	1.00 (0.98, 1.02)	0.97 (0.96, 0.99)
	4	110059(84.6%)	22543(15.4%)	1.11 (1.09, 1.13)	1.03 (1.01, 1.05)
	5 – most deprived	109248(85.5%)	21450(14.5%)	1.16 (1.14, 1.18)	1.03 (1.01, 1.05)
Confidence in managing health	Very confident	240196(82.3%)	58806(17.7%)	Reference	Reference
	Fairly confident	283776(84.9%)	58435(15.1%)	1.19 (1.17, 1.20)	1.21 (1.19, 1.22)
	Not very confident	33166(89.1%)	4562(10.9%)	1.78 (1.72, 1.84)	1.75 (1.70, 1.80)
	Not at all confident	5509(88.2%)	794(11.8%)	1.70 (1.58, 1.83)	1.73 (1.61, 1.85)
Long term condition	No	216594(85.2%)	42250(14.8%)	Reference	Reference
	Yes	346053(83.0%)	80347(17.0%)	0.84 (0.83, 0.85)	0.97 (0.96, 0.99)

† *Weighted percentages are calculated using survey design and non-response weights (by age, gender, geographical location and GP practice, full details Technical Annex [13])
*p<0.001 for all except gender where p=0.969 **p<0. 001 for all except gender where p = 0. 024 ‡OR prefer nurse come from the same model as prefer GP and are equal to 1/(OR prefer GP)

Table 2. Associations between patient characteristics and wanting an appointment to see or speak to a GP. Odds ratios > 1 indicate patient group more likely to want to see a GP than the reference group. Only those with complete information for all covariates are included (n=728,976)

For peer review only

		N (%)	Patient-rated confidence and trust	Crude difference* (95% CI)	Adjusted difference** (95% CI)
Consultation outcome: saw/spoke to nurse					
Patient request	Appointment outcome				
See a nurse	Saw a nurse	105713 (87.0%)	91.0	Reference	Reference
See a nurse	Spoke to a nurse	518 (0.4%)	87.6	-3.40 (-5.27, -1.53)	-2.87 (-4.70, -1.04)
Speak to a nurse	Saw a nurse	1165 (1.0%)	87.8	-3.23 (-4.48, -1.98)	-2.82 (-4.05, -1.60)
Speak to a nurse	Spoke to a nurse	1698 (1.4%)	88.3	-2.70 (-3.74, -1.66)	-2.40 (-3.42, -1.38)
See a GP	Saw a nurse	10769 (8.9%)	79.5	-11.58 (-12.01, -11.15)	-9.90 (-10.33, -9.47)
See a GP	Spoke to a nurse	534 (0.4%)	72.9	-18.10 (-19.94, -16.26)	-15.83 (-17.64, -14.02)
Speak to a GP	Saw a nurse	821 (0.7%)	83.9	-7.18 (-8.67, -5.69)	-6.04 (-7.50, -4.58)
Speak to a GP	Spoke to a nurse	286 (0.2%)	76.0	-14.99 (-17.51, -12.48)	-13.47 (-15.93, -11.01)
Consultation outcome: saw/spoke to GP					
Patient request	Appointment outcome				
See a GP	Saw a GP	446631 (90.6%)	84.4	Reference	Reference
See a GP	Spoke to a GP	9000 (1.8%)	76.7	-7.68 (-8.22, -7.13)	-6.47 (-7.00, -5.93)
Speak to a GP	Saw a GP	8190 (1.7%)	83.6	-0.82 (-1.40, -0.25)	-0.77 (-1.33, -0.22)
Speak to a GP	Spoke to a GP	21959 (4.5%)	86.4	1.96 (1.61, 2.32)	1.42 (1.07, 1.76)
See a nurse	Saw a GP	5763 (1.2%)	84.2	-0.19 (-0.87, 0.49)	-0.73 (-1.39, -0.08)
See a nurse	Spoke to a GP	370 (0.1%)	79.2	-5.21 (-7.88, -2.54)	-5.63 (-8.20, -3.07)
Speak to a nurse	Saw a GP	893 (0.2%)	82.9	-1.48 (-3.20, 0.24)	-1.48 (-3.13, 0.18)
Speak to a nurse	Spoke to a GP	173 (0.1%)	80.6	-3.76 (-7.67, 0.14)	-4.04 (-7.80, -0.29)

*p<0.001 (joint test)

**Also adjusted for age, gender, ethnicity, confidence in managing their own health, the presence of a long standing heath condition, deprivation (fixed effects) and practice (random effect) p<0.001 (joint test)

Table 3. Results of the regression analyses examining the association between patient-rated confidence and trust in health professionals, and the difference in the type of appointment patients wanted and the appointment they got at their last consultation in primary care.

For peer review only

For peer review only - http://bmjopen.bmj.com/site/about/guidelines.xhtml

		N (%)	Mean Communication Score	Crude difference* (95% CI)	Adjusted difference** (95% CI)
Consultation outcome: saw/spoke to nurse					
Patient request	Appointment outcome				
See a nurse	Saw a nurse	105140 (86.8%)	90.0	Reference	Reference
See a nurse	Spoke to a nurse	517 (0.4%)	88.3	-1.70 (-2.97, -0.44)	-1.40 (-2.63, -0.18)
Speak to a nurse	Saw a nurse	1170 (1.0%)	87.6	-2.37 (-3.21, -1.53)	-2.05 (-2.87, -1.23)
Speak to a nurse	Spoke to a nurse	1697 (1.4%)	88.9	-1.09 (-1.79, -0.38)	-0.93 (-1.61, -0.24)
See a GP	Saw a nurse	10916 (9.0%)	82.7	-7.31 (-7.60, -7.02)	-5.99 (-6.28, -5.71)
See a GP	Spoke to a nurse	538 (0.4%)	77.8	-12.16 (-13.40, -10.92)	-10.47 (-11.68, -9.27)
Speak to a GP	Saw a nurse	819 (0.7%)	85.7	-4.25 (-5.26, -3.25)	-3.35 (-4.33, -2.38)
Speak to a GP	Spoke to a nurse	289 (0.2%)	80.2	-9.80 (-11.49, -8.11)	-8.66 (-10.30, -7.03)
Consultation outcome: saw/spoke to GP					
Patient request	Appointment outcome				
See a GP	Saw a GP	450555 (90.6%)	85.4	Reference	Reference
See a GP	Spoke to a GP	9127 (1.8%)	80.0	-5.41 (-5.77, -5.05)	-4.49 (-4.84, -4.14)
Speak to a GP	Saw a GP	8281 (1.7%)	85.2	-0.21 (-0.59, 0.17)	-0.23 (-0.59, 0.13)
Speak to a GP	Spoke to a GP	22039 (4.4%)	86.9	1.54 (1.30, 1.77)	0.96 (0.73, 1.19)
See a nurse	Saw a GP	5831 (1.2%)	84.5	-0.85 (-1.30, -0.40)	-1.22 (-1.65, -0.80)
See a nurse	Spoke to a GP	378 (0.1%)	82.0	-3.41 (-5.17, -1.64)	-3.76 (-5.43, -2.10)
Speak to a nurse	Saw a GP	913 (0.2%)	84.3	-1.07 (-2.21, 0.07)	-0.94 (-2.01, 0.13)
Speak to a nurse	Spoke to a GP	178 (0.0%)	83.7	-1.64 (-4.21, 0.93)	-2.16 (-4.59, 0.26)

*p<0.001 (joint test)

**Also adjusted for age, gender, ethnicity, confidence in managing their own health, the presence of a long standing heath condition, deprivation (fixed effects) and practice (random effect) p<0.001 (joint test)

Table 4 – Results of regression analyses examining the association between patient rated nurse and GP communication scores, and the difference in the type of appointment patients wanted and the appointment they got at their last consultation in primary care.

For peer review only

For peer review only - http://bmjopen.bmj.com/site/about/guidelines.xhtml

Appendix Table 1. Five category summary of responses to the question “What did you want to do?” in the GP Patient Survey

	Response options to question: Last time you wanted to see or speak to a GP or nurse from your GP surgery: What did you want to do?*					
	See a GP at the surgery	See a nurse at the surgery	Speak to a GP on the phone	Speak to a nurse on the phone	Have someone visit me at my home	I didn’t mind / wasn’t sure what I wanted
Wanted to see or speak to a GP (or both)	X					
			X			
	X		X			
Wanted to see or speak to a Nurse (or both)		X				
				X		
		X		X		
Wanted a home visit					X	
Wasn’t sure or didn’t mind						X
Wanted more than one of the previous four categories	Any Other combination					

*Response options available to primary care patients completing the 2013/14 General Practice Patient Survey (http://www.gp-patient.co.uk).

For peer review only

For peer review only - http://bmjopen.bmj.com/site/about/guidelines.xhtml

Appendix Table 2. Two category summary of responses to the question “What did you want to do?” in the GP Patient Survey

	Last time you wanted to see or speak to a GP or nurse from your GP surgery: What did you want to do?					
	See a GP at the surgery	See a nurse at the surgery	Speak to a GP on the phone	Speak to a nurse on the phone	Have someone visit me at my home	I didn’t mind / wasn’t sure what I wanted
Wanted to see or speak to a GP (or both)	X					
			X			
	X		X			
	X					X*
			X			X*
	X		X			X*
Wanted to see or speak to a Nurse (or both)		X				
				X		
		X		X		
		X				X*
				X		X*
		X		X		X*
Excluded	Any Other combination					

*Where a patient indicated they didn’t mind while only indicating an appointment with either a GP or nurse, but not both, we took this to indicate they did not mind how they interacted with the healthcare professional, but they had expressed a preference for a particular type of health professional

For peer review only

For peer review only

For peer review only - http://bmjopen.bmj.com/site/about/guidelines.xhtml

Checklists

A STROBE checklist is included as a supplementary file.

Exclusive license

The Corresponding Author has the right to grant on behalf of all authors and does grant on behalf of all authors, a worldwide license to the Publishers and its licensees in perpetuity, in all forms, formats and media (whether known now or created in the future), to i) publish, reproduce, distribute, display and store the Contribution, ii) translate the Contribution into other languages, create adaptations, reprints, include within collections and create summaries, extracts and/or, abstracts of the Contribution, iii) create any other derivative work(s) based on the Contribution, iv) to exploit all subsidiary rights in the Contribution, v) the inclusion of electronic links from the Contribution to third party material where-ever it may be located; and, vi) license any third party to do any or all of the above.

Contributorship statement

All authors (CP, GA, JB, JC, ME, VL, and MR) jointly conceived of the research questions, reviewed/edited the manuscript and contributed to discussion and interpretation of the data. CP and GA led on writing the manuscript, and GA completed the statistical analysis. All authors (CP, GA, JB, JC, ME, VL, and MR) had full access to all of the data in the study and take responsibility for the integrity of the data and accuracy of the data analysis. CP is the guarantor of the paper.

Ethical approval

The study did not require ethical approval

Competing interests

We have read and understood BMJ Open policy on declaration of interests and declare that we have no competing interests.

Data sharing

No additional data are available.

Transparency declaration.

Dr Paddison affirms that the manuscript is an honest, accurate, and transparent account of the study being reported; that no important aspects of the study have been omitted. There are no significant discrepancies from the study as planned.

Funding

This paper presents independent research funded by the National Institute for Health Research (NIHR) under its Programme Grants for Applied Research Programme (Grant Reference Number RP-PG-0608-10050). The views expressed are those of the author(s) and not necessarily those of the NHS, the NIHR or the Department of Health. The researchers confirm their independence from the study funders, the National Institute for Health Research.

For peer review only

For peer review only - http://bmjopen.bmj.com/site/about/guidelines.xhtml

Open Access statement

This is an open access article distributed in accordance with the terms of the Creative Commons Attribution (CC BY 4.0) license, which permits others to distribute, remix, adapt and build upon this work, for commercial use, provided the original work is properly cited. See: http://creativecommons.org/licenses/by/4.0

Sponsorship

The study was sponsored by the University of Cambridge.

References

[1] Campbell SM, Roland MO, Buetow SA. Defining quality of care. Social Science & Medicine. 2000;51(11):1611-25. http://www.sciencedirect.com/science/article/pii/S0277953600000575

[2] Department of Health. The NHS Outcomes Framework 2011/12 2010 www.dh.gov.uk/en/Publicationsandstatistics/Publications/PublicationsPolicyAndGuidance/DH_122944

[3] Department of Health. Equity and Excellence: Liberating the NHS 2010. https://www.gov.uk/government/publications/liberating-the-nhs-white-paper

[4] House of Commons Health Committee. Primary Care: Fourth Report of Session 2015–16. www.publications.parliament.uk/pa/cm201516/cmselect/cmhealth/408/408.pdf

[5] Roland M, Everington S. Tackling the crisis in general practice. BMJ. 2016;352. http://www.bmj.com/content/352/bmj.i942

[6] Primary Care Workforce Commission. The future of primary care: Creating teams for tomorrow. 2015. Health Education England https://www.hee.nhs.uk/our-work/hospitals-primary-community-care/primary-community-care/primary-care-workforce-commission

[7] NHS England. General Practice Forward View. 2016. https://www.england.nhs.uk/wp-content/uploads/2016/04/gpfv.pdf

[8] Horrocks S, Anderson E, Salisbury C. Systematic review of whether nurse practitioners working in primary care can provide equivalent care to doctors. BMJ. 2002;324(7341):819-23. http://www.bmj.com/content/324/7341/819

[9] Laurant M, Reeves D, Hermens R, Braspenning J, Grol R, Sibbald B. Substitution of doctors by nurses in primary care. Cochrane Database of Systematic Reviews. 2005. http://onlinelibrary.wiley.com/doi/10.1002/14651858.CD001271.pub2/pdf/

[10] Hegney D, Price K, Patterson E, Martin-McDonald K, Rees S. Australian consumers' expectations for expanded nursing roles in general practice - choice not gatekeeping. Australian Family Physician. 2004;33(10):845-9 http://www.racgp.org.au/afpbackissues/2004/200409/20040930hegney.pdf

For peer review only - http://bmjopen.bmj.com/site/about/guidelines.xhtml

[11] Redsell S, Stokes T, Jackson C, Hastings A, Baker R. Patients' accounts of the differences in nurses' and general practitioners' roles in primary care. Journal of Advanced Nursing. 2007;57(2):172-80. http://onlinelibrary.wiley.com/doi/10.1111/j.1365-2648.2006.04085.x/full

[12] Campbell J, Smith P, Nissen S, Bower P, Elliott M, Roland M. The GP Patient Survey for use in primary care in the National Health Service in the UK - development and psychometric characteristics. BMC Family Practice. 2009;10(1):57. http://bmcfampract.biomedcentral.com/articles/10.1186/1471-2296-10-57

[13] Ipsos MORI. GP Patient Survey - Technical annex. 2016. https://gp-patient.co.uk/downloads/archive/2016/July/July%202016%20National%20Technical%20Report.pdf

[14] Lyratzopoulos G, Elliott M, Barbiere J, Henderson A, Staetsky L, Paddison C, Campbell J, Roland M. Understanding ethnic and other socio-demographic differences in patient experience of primary care: evidence from the English General Practice Patient Survey. BMJ Quality and Safety 2012; 21: 21-9 http://qualitysafety.bmj.com/content/21/1/21

[15] Roberts M, Campbell J, Abel G, Davey A, Elmore N, Maramba I, Carter M, Elliott M, Roland M, Burt J. Understanding high and low patient experience scores in primary care: analysis of patients' survey data for general practices and individual doctors. BMJ 2014; 349: g6034 http://www.bmj.com/content/349/bmj.g6034

[16] Lyratzopoulos G, Barbiere J, Elliott M, Staestky L, Paddison C, Campbell J, et al. How can healthcare providers be reliably compared? Lessons from a national survey of patient experience . Medical Care 2011;49(8):724-33. https://www.jstor.org/stable/23053838?seq=1#page_scan_tab_contents

[17] Campbell JL, Fletcher E, Britten N, Green C, Holt TA, Lattimer V, et al. Telephone triage for management of same-day consultation requests in general practice (the ESTEEM trial): a cluster-randomised controlled trial and cost-consequence analysis. Lancet.384(9957):1859-68. http://www.thelancet.com/journals/lancet/article/PIIS0140-6736(14)61058-8/abstract

[18] Donelan K, DesRoches CM, Dittus RS, Buerhaus P. Perspectives of Physicians and Nurse Practitioners on Primary Care Practice. New England Journal of Medicine. 2013;368(20):1898-906. http://www.nejm.org/doi/full/10.1056/NEJMsa1212938#t=article

For peer review only

For peer review only - http://bmjopen.bmj.com/site/about/guidelines.xhtml

STROBE Statement—checklist of items that should be included in reports of observational studies

	Item No.	Recommendation	Page No.	Relevant text from manuscript
**Title and abstract**	1	(*a*) Indicate the study's design with a commonly used term in the title or the abstract	1	Study type is in the title and abstract
		(*b*) Provide in the abstract an informative and balanced summary of what was done and what was found	1	Abstract
		**Introduction**		
Background/rationale	2	Explain the scientific background and rationale for the investigation being reported	2	Background section
Objectives	3	State specific objectives, including any pre-specified hypotheses	2	Two objectives are specified.
		**Methods**		
Study design	4	Present key elements of study design early in the paper	2-3	Study design is in both the background and the method section.
Setting	5	Describe the setting, locations, and relevant dates, including periods of recruitment, exposure, follow-up, and data collection	2-3	Method section
Participants	6	(*a*) *Cohort study*—Give the eligibility criteria, and the sources and methods of selection of participants. Describe methods of follow-up		N/A
		Case-control study—Give the eligibility criteria, and the sources and methods of case ascertainment and control selection. Give the rationale for the choice of cases and controls		N/A
		Cross-sectional study—Give the eligibility criteria, and the sources and methods of selection of participants	2-3	Selection of patients to include described in method section.
		(*b*) *Cohort study*—For matched studies, give matching criteria and number of exposed and unexposed		N/A
		Case-control study—For matched studies, give matching criteria and the number of controls per case		N/A
Variables	7	Clearly define all outcomes, exposures, predictors, potential confounders, and effect modifiers. Give diagnostic criteria, if applicable	2-3	Outcome s are specified questions in the GP Patient Survey
Data sources/	8*	For each variable of interest, give sources of data and details of methods of assessment	2,5	We report on the response rate

For peer review only - http://bmjopen.bmj.com/site/about/guidelines.xhtml

measurement		(measurement). Describe comparability of assessment methods if there is more than one group		to the survey and published evidence that this is unlikely to be associated with major biases in the data
Bias	9	Describe any efforts to address potential sources of bias	2-3	We adjusted for major potential confounders. Other details are referenced to our previous approach to analysing these data (references 14 and 15) and to the IPSOS MORI Technical Annex to the GP Patient Survey (reference 13)
Study size	10	Explain how the study size was arrived at	2-3	We used all data that met our eligibility criteria from respondents to one year of the GP Patient Survey (870.085 respondents). There was no formal sample size calculation: with these numbers, statistical significance is likely to be reached for very small effects. Of greater relevance is the interpretation of small but statistically significant differences.

Continued on next page

For peer review only - http://bmjopen.bmj.com/site/about/guidelines.xhtml

For peer review only

Quantitative variables	11	Explain how quantitative variables were handled in the analyses. If applicable, describe which groupings were chosen and why	2-3	See responses to 6,7 and 9 above.
Statistical methods	12	(*a*) Describe all statistical methods, including those used to control for confounding	2-3	Potential confounders are listed in the method section and included as adjusted estimates of effect size (which are the main ones we report).
		(*b*) Describe any methods used to examine subgroups and interactions		As above
		(*c*) Explain how missing data were addressed		See answer to question 9. These references (14 and 15) also describe how we dealt with patients who did not complete all items in the communication section of the GP Patient Survey.
		(*d*) *Cohort study*—If applicable, explain how loss to follow-up was addressed		N/A
		Case-control study—If applicable, explain how matching of cases and controls was addressed		N/A
		Cross-sectional study—If applicable, describe analytical methods taking account of sampling strategy	2-3	We describe how we used survey weights to account for survey design and non-response.
		(*e*) Describe any sensitivity analyses		None
**Results**				
Participants	13*	(a) Report numbers of individuals at each stage of study—eg numbers potentially eligible, examined for eligibility, confirmed eligible, included in the study, completing follow-up, and analysed	4	Results section
		(b) Give reasons for non-participation at each stage		N/A
		(c) Consider use of a flow diagram		Not required
Descriptive data	14*	(a) Give characteristics of study participants (eg demographic, clinical, social) and information on exposures and potential confounders	8	Listed in table 2 – these were included in the adjusted estimates in tables 3 and 4.
		(b) Indicate number of participants with missing data for each variable of interest	4	We used only subjects with complete data (n=685,244)
		(c) *Cohort study*—Summarise follow-up time (eg, average and total amount)		N/A

For peer review only - http://bmjopen.bmj.com/site/about/guidelines.xhtml

Outcome data	15*	*Cohort study*—Report numbers of outcome events or summary measures over time		N/A
		Case-control study—Report numbers in each exposure category, or summary measures of exposure		N/A
		Cross-sectional study—Report numbers of outcome events or summary measures	2-4	Two main outcomes as described in the introduction (objectives) and method sections.
Main results	16	(*a*) Give unadjusted estimates and, if applicable, confounder-adjusted estimates and their precision (eg, 95% confidence interval). Make clear which confounders were adjusted for and why they were included	9-10	Tables 3 and 4 give both unadjusted (crude) and adjusted estimates.
		(*b*) Report category boundaries when continuous variables were categorized		N/A
		(*c*) If relevant, consider translating estimates of relative risk into absolute risk for a meaningful time period		N/A

Continued on next page

For peer review only

Other analyses	17	Report other analyses done—eg analyses of subgroups and interactions, and sensitivity analyses		No other analyses
**Discussion**				
Key results	18	Summarise key results with reference to study objectives	5	Discussion
Limitations	19	Discuss limitations of the study, taking into account sources of potential bias or imprecision. Discuss both direction and magnitude of any potential bias	5	Discussion
Interpretation	20	Give a cautious overall interpretation of results considering objectives, limitations, multiplicity of analyses, results from similar studies, and other relevant evidence	5-6	Discussion
Generalisability	21	Discuss the generalisability (external validity) of the study results	5	Discussed in terms of this being a strength of the study, as it uses a large national survey with data from over 8000 general practices (i.e. almost all those in England)
**Other information**				
Funding	22	Give the source of funding and the role of the funders for the present study and, if applicable, for the original study on which the present article is based	14	The study was funded by the National Institute for Health Research (programme grant RP-PG-0608-10050)

*Give information separately for cases and controls in case-control studies and, if applicable, for exposed and unexposed groups in cohort and cross-sectional studies.

Note: An Explanation and Elaboration article discusses each checklist item and gives methodological background and published examples of transparent reporting. The STROBE checklist is best used in conjunction with this article (freely available on the Web sites of PLoS Medicine at http://www.plosmedicine.org/, Annals of Internal Medicine at http://www.annals.org/, and Epidemiology at http://www.epidem.com/). Information on the STROBE Initiative is available at www.strobe-statement.org.

For peer review only - http://bmjopen.bmj.com/site/about/guidelines.xhtml

BMJ Open

What happens to patient experience when you want to see a doctor and you get to speak to a nurse? Observational study using data from the English General Practice Patient Survey

Journal:	*BMJ Open*
Manuscript ID	bmjopen-2017-018690.R1
Article Type:	Research
Date Submitted by the Author:	30-Nov-2017
Complete List of Authors:	Paddison, Charlotte; Nuffield Trust, ; University of Cambridge, Institute of Public Health<br>Abel, Gary; University of Exeter, University of Exeter Medical School (Primary Care)<br>Burt, Jenni; University of Cambridge, Cambridge Centre for Health Services Research<br>Campbell, John; University of Exeter, Primary Care;<br>Elliot, Marc; RAND corporation,<br>Lattimer, Valerie; UEA, School of Nursing Sciences<br>Roland, Martin; Institute of Public Health, Institute of Public Health
**Primary Subject Heading**:	General practice / Family practice
Secondary Subject Heading:	Health services research, Health policy
Keywords:	Health services research, PRIMARY CARE, patient experience, Quality in health care < HEALTH SERVICES ADMINISTRATION & MANAGEMENT

SCHOLARONE™
Manuscripts

Title:

What happens to patient experience when you want to see a doctor and you get to speak to a nurse? Observational study using data from the English General Practice Patient Survey

Authors:

Charlotte A. M. Paddison PhD [1], Gary A. Abel PhD [2], Jenni Burt PhD [3], John Campbell MD FRCGP [2], Marc N. Elliott PhD [4], Valerie Lattimer [5], Martin Roland DM FRCGP [3]

[1] Nuffield Trust, 59 New Cavendish Street, London, UK W1G 7LP

[2] University of Exeter Medical School, St Lukes Campus, Magdalen Road, Exeter EX1 2LU, UK

[3] Cambridge Centre for Health Services Research, Primary Care Unit, Institute of Public Health, Forvie Site, University of Cambridge School of Clinical Medicine, Box 113 Cambridge Biomedical Campus, Cambridge, CB2 0SR, UK

[4] RAND Corporation, 1776 Main Street, P.O. Box 2138, Santa Monica, CA 90407-2138

[5] University of East Anglia, School of Nursing Science, Norwich Research Park, Norwich, Norfolk UK NR4 7TJ

Corresponding author

Charlotte Paddison

Nuffield Trust, 59 New Cavendish Street, London, UK W1G 7LP

Phone: 0207 462 0548

Email: (academic) camp3@medschl.cam.ac.uk

For peer review only

Secondary email: (non-academic/employer) charlotte.paddison@nuffieldtrust.org.uk

Keywords

patient experience, health services research, primary care, health care quality

Word count (manuscript, excluding title page, abstract, references, figures): 2,734

For peer review only

Abstract

Objectives: To examine patient consultation preferences for seeing or speaking to a GP or nurse; to estimate associations between patient-reported experiences and the type of consultation patients actually received (phone or face-to-face, GP or nurse)

Design: Secondary analysis of data from the 2013/14 GP Patient Survey

Setting and participants: 870,085 patients from 8,005 English general practices

Outcomes: Patient ratings of communication and 'trust and confidence' with the clinician they saw.

Results: 77.7% of patients reported wanting to see or speak to a GP, while 14.5% reported asking to see or speak to a nurse the last time they tried to make an appointment (weighted percentages). Being unable to see or speak to the practitioner type of the patients' choice was associated with lower ratings of trust and confidence and patient-rated communication. Smaller differences were found if patients wanted a face-to-face consultation and received a phone consultation instead. The greatest difference was for patients who asked to see a GP and instead spoke to a nurse for whom the adjusted mean difference in confidence and trust compared to those who wanted to see a nurse and did see a nurse was -15.8 points (95% CI -17.6, -14.0) for confidence and trust in the practitioner and -10.5 points (95%CI -11.68, -9.27) for net communication score, both on a 0-100 scale.

Conclusions: Patients' evaluation of their care is worse if they do not receive the type of consultation they expect, especially if they prefer a doctor but are unable to see one. New models of care should consider the potential unintended consequences for patient experience of the widespread introduction of multi-disciplinary teams in general practice.

(Word count: 272 words)

Strengths and limitations of this study

- Large national survey with respondents including 870,085 patients from 8,005 English general practices
- Appointment choice (mode and type of practitioner), and patient-reported experience, all collected in a single survey.
- Thirty-four per cent response rate, though typical for similar national surveys.
- Patient-reported confidence and trust assessed using a single item within the GP Patient Survey.

For peer review only - http://bmjopen.bmj.com/site/about/guidelines.xhtml

For peer review only

Patient experience is a core dimension of health care quality[1] and measuring patient experience enables the views of service users to be incorporated into the evaluation and improvement of health services at local and national level.[2,3] In the UK, 90% of all NHS patient contacts occur in primary care. Though primary care is highly valued by the UK public, the primary care workforce is under unprecedented strain[4,5] and clear policies for reforming primary care in England are now emerging.[6,7] These include the development of wider multi-disciplinary teams that make greater use of nurses, physicians' associates, pharmacists, and administrative support. It is anticipated that an expanded workforce will free up clinical time for doctors, enabling them to devote more time to the delivery of care for complex patients and spend less time on administration and minor health issues[7].

At present it is unclear what impact these proposed changes to the primary care workforce might have on patient choice and quality of care, including patient experience. Multi-disciplinary teams could increase patient choice by improving access to nurses and pharmacists, but their introduction could also have unintended adverse impacts, for example on patients who were no longer able to see the type of clinician of their choice.

Given the policy emphasis on promoting patient choice, evidence is needed on the acceptability to patients of seeing different practitioners, especially when their original intention had been to see a GP. Systematic reviews suggest that nurse-GP substitution in primary care can produce similar or better quality of care and high levels of patient satisfaction[8,9], but there is also evidence that some patients do not want nurses to substitute for GPs.[10] Relational continuity of care, together with perceived trust and confidence, have emerged as important factors in understanding why some patients may prefer to see a GP and, more specifically, a GP who knows them well.[11]

In this primary care-based study we examine patient consultation preferences and their association with patient-reported experiences, using data from the English General Practice Patient Survey (GPPS) to address the following questions:

1. To what extent do patients in primary care request an appointment with a doctor or a nurse, and how often is this request met?

2. When patients are unable to get an appointment of their choice (phone or face-to-face, GP or nurse), is this associated with poorer patient-reported communication or lower trust and confidence?

Methods

We analysed data from the 2013/14 General Practice Patient Survey (http://www.gp-patient.co.uk), which is sent as a mail questionnaire to 2.6 million patients in England each year[12]. We estimated preference for nurse and GP appointments from the question which asked respondents 'Last time you wanted to see or speak to a GP or nurse from your GP surgery: What did you want to do?' with response options coded into five categories (see Appendix Table 1):

- see or speak to a GP
- see or speak to a Nurse

For peer review only - http://bmjopen.bmj.com/site/about/guidelines.xhtml

- a home visit
- wasn't sure or didn't mind
- wanted more than one of the previous four categories

Practice population estimates were computed using survey weights that account for survey design and non-response.[13] Variation in preferences for GP and nurse appointments were explored using crude and adjusted logistic regression using a binary classification (wanting to see or speak to a nurse vs. wanting to see or speak to a GP as the outcome - see Appendix Table 2 for detail of how categories were constructed), excluding those indifferent as to whether they saw a doctor or a nurse. Fixed effects included in the models were patient reported age, gender, ethnicity, confidence in managing their own health, and presence of a long-term condition, as well as a measure of social deprivation based on the patient's postcode of residence (national quintiles as categories). General practice was included as a random effect (intercept). All analyses were restricted to patients with complete information on all variables included in the adjusted model.

Next we investigated whether people who did not get an appointment with the type of practitioner of their choice were more likely to report poorer patient experience. For simplicity this analysis was restricted to those who only endorsed one response option each for the question on what type of appointment they had requested and for the question on what they got. Sixteen categories, combinations of seeing or speaking to a health professional and whether the appointment was with a GP or a nurse, were created. The dataset was then split into those whose last appointment was with a GP and those whose last appointment was with a nurse, and these were modelled separately.

Two series of regression models were run. The first series of models used responses to the question 'Did you have confidence and trust in the GP (nurse) you saw or spoke to?' as the outcome. Response options were: 'yes, definitely'; 'yes, to some extent'; and 'no, not at all' which were scored as 100, 50 and 0 respectively. Crude differences between the categories were estimated for trust and confidence in GPs and, separately, for nurses, using linear regression. Mixed-effect linear regression was then used for an adjusted analysis including age, gender, ethnicity, confidence in managing their own health, the presence of a long standing heath condition, and deprivation. Practice was included as a random effect (intercept), and analyses were restricted to those who had complete information on all socio-demographic and health variables in this study.

A second series of models using the same approach (linear regressions to estimate crude differences between categories, and mixed-effect linear regression for analyses adjusting for health and demographic characteristics and additionally controlling for practice) were then run using composite patient-rated communication scores as the outcome. This composite score was the mean of all responses to five questions on patient reported experiences of communication for patients who answered at least three of these questions. Scores were computed separately for doctor communication (five items) and nurse communication (five items). The questions asked how good the GP or nurse was at 'giving you enough time', 'listening to you' or 'explaining tests and treatments', 'involving you in decisions about your care and 'treating you with care and concern'. As in our previous work, responses to these patient experience items were linearly rescaled to give a score from 0 to 100 as in previous work in order to facilitate comparison between items.[14,15]

For peer review only - http://bmjopen.bmj.com/site/about/guidelines.xhtml

Results

Among 870,085 primary care patients from 8005 practices in England, more than three quarters reported that they only asked to see or speak to a GP, whilst just under 15% only asked to see or speak to a nurse (Table 1) with the remainder wanting a home visit, expressing no preference or asking for multiple types of appointment. Comparing the type of appointment patients wanted and the type of appointment they actually received, we found that the great majority of people got what they wanted. As shown in Table 1, 96% of people who wanted to see or speak to a GP achieved this, compared to 92% of people who wanted to see or speak to a nurse. The number wanting a home visit who got one was lower at 80%.

<insert Table 1 approximately here>

There were 685,244 respondents who only wanted to see or speak to either a nurse or doctor who also had complete information for all covariates of interest. Of these 122,597 (weighted percentage 16.0%) wanted to see or speak to a nurse, with the remainder wanting to see or speak to a GP. Variation in this percentage is shown in Table 2 along with crude and adjusted odds ratios. Older patients were more likely to want to see or speak to a nurse than younger patients (e.g. 85 and over vs. 55 to 64 year olds adjusted OR=1.31 95% CI 1.26, 1.36). Ethnic minority group patients were much less likely to want to see or speak to a nurse (e.g. Asian vs. White patients adjusted OR=0.66 95% CI 0.64, 0.68). Similarly those who were not confident in managing their own health were less likely to want to see or speak to a nurse (e.g. Not at all confident vs. Very confident adjusted OR=0.58 95% CI 0.54, 0.62).

<insert Table 2 approximately here>

Being unable to get an appointment of the patients' choice was associated with differences in trust and confidence (Table 3) and communication (Table 4), among patients who reported seeing or speaking to a nurse or a GP in their last primary care consultation. The mean trust and confidence score for those who had asked to see a nurse and did so was 91.0 (on a scale of 0- 100). For all other combinations patient-rated trust and confidence scores were, on average, lower ($p<0.001$). Patient reported trust and confidence was lowest for those who wished to see a GP or wished to speak to a GP, and instead spoke to a nurse (adjusted mean difference compared to those who wanted to see a nurse and did see a nurse = -15.8 95% CI -17.6, -14.0, and -13.5 95% CI -15.9, -11.0, respectively), followed by those who wanted to see a GP but ended up speaking to, rather than seeing, a GP (-6.5, 95% CI -7.0, -5.9). Similar differences were found for patient reported communication scores, with

For peer review only - http://bmjopen.bmj.com/site/about/guidelines.xhtml

the lowest scores for those who wanted to see a GP but spoke to a nurse (adjusted mean difference compared to those who wanted to see a nurse and did see a nurse = -10.5 95%CI -11.7, -9.2).

<insert Table 3 approximately here>

<insert Table 4 approximately here>

Discussion

In a study of 870,085 respondents from general practices in England, we show that the majority of patients in primary care are able to see or speak to the health professional type (GP vs nurse) of their choice. However a substantive minority - between four per cent and eight per cent - are not. We found evidence that patient consultation preferences vary across patient groups. Patients with low confidence in managing their own health, younger patients and ethnic minority groups were more likely to want to see a GP.

When patients were unable to obtain the type of appointment of their choice, this was associated with lower confidence and trust in the health professional they saw, and poorer patient-rated communication. This difference was particularly large among patients who wished to see a GP and instead spoke to a nurse. A difference, though smaller in magnitude, was also observed for patients who wanted to see a nurse and got to speak to a GP. Patients whose appointments were with GPs reported similar experiences regardless of their initial preference for a nurse versus a doctor. Patient experience scores were also lower when the patient had asked to see a health professional (either a GP or a nurse), and ended up having a telephone call.

Our study, using data from a large national survey of patient experience in primary care, builds on what is known from previous research examining the acceptability to patients' of nurse-GP substitution[8,9,16,17] and extends on smaller qualitative studies of patients' preferences for GP versus nurse-led consultations[10,11], controlled trials investigating the impacts of nurse-led primary care on hospital admission and mortality[18], and qualitative synthesis of evidence on the barriers and facilitators to the implementation of doctor-nurse substitution strategies in primary care[19]. In particular, this study highlights the possible unintended impacts of nurse-GP substitution on patients' confidence and trust, and the quality of communication in primary care. The response rate (34%) in our study is comparable with other major national surveys, and a previous assessment found no association between practice response rates and scores[20] Despite its large overall sample, one limitation of our study is the smaller size of some individual patient subgroups giving rise to larger uncertainty. Another limitation is that we assessed patient-reported confidence and trust using the single item that represents this concept in the GP Patient Survey, and future research might consider including additional items to provide a comprehensive and multidimensional measure of these constructs.

Changing nature of primary care and implications for patient confidence and trust

For peer review only - http://bmjopen.bmj.com/site/about/guidelines.xhtml

The role of nurses in primary care has changed considerably in the last 30 years, with the advent of advanced nurse practitioners, independent nurse prescriber roles, and the existence of some primary care practices that are led by nurses. Some patients appear uncertain about what to expect in nurse-led primary care consultations.[9,11] In our study we found that patients who got to speak to a nurse when they wished to see a GP, reported lowest confidence and trust in their eventual consultation. The magnitude of this effect represents a large difference of 15 points on a scale of 0 – 100, controlling for socio-demographic characteristics and health status and combines not seeing or speaking to the type of practitioner of their choice (i.e. nurse rather than doctor) and receiving a telephone call when they had asked for a face to face consultation. There are several possible explanations for these findings. Patients may feel that they are being inappropriately denied access to GPs, resulting in frustration that could affect both trust and the quality of interpersonal communication (*gatekeeper hypothesis*). Lack of trust and confidence may also arise because some patients perceive that nurses are subordinate to GPs in terms of skills and knowledge (*patient confidence hypothesis*). Our findings are in line with the results from a pragmatic controlled trial of telephone triage among patients seeking a same-day GP appointment which showed that nurse-led triage was associated with somewhat worse patient-reported experience and lower overall patient satisfaction in comparison with usual care.[21]

Implications for health policy, clinical practice and primary care research

A strong vision for reforming primary care in England is now emerging.[6] This includes expanded roles for pharmacists, physician associates, and a £15 million investment in nursing capacity within General Practice. When new ways of working are introduced, primary care practices need to communicate these to patients who may know less about the care offered by health professionals other than GPs. Building patient confidence and educating patients about the skills of team members should form part of a broader effort to involve patients in the design and development of new services. Involving patients in service redesign may help to address barriers that affect patient willingness to consult with nurses or physician associates, for example patient-reported experiences of incomplete or delayed care (including prescription delay), and concerns about the loss of provider continuity[22].

In developing new models of working, staff training needs to be carefully considered. A study of nurse-led clinics for patients with osteoarthritis in general practice found a significant gap between NICE recommendations and the care nurses felt confident and able to deliver[23]. Staff transitioning in to new roles in patient care or in patient triage need to feel adequately prepared, and standardised training may be necessary, but insufficient, to ensure success[24]. New models of care should consider the possible intended and unintended consequences for patient experience of multi-disciplinary teams, particularly given the policy emphasis on enabling patient choice. Monitoring should include any potential adverse effects on patient confidence and trust in health professionals. Qualitative interviews with patients' consulting a physician associate in general practice highlight a desire for continuity with a trusted clinician, the importance of patient choice, and the maintenance of trust and confidence in general practice[22].

In the context of aging populations, general practices are seeing increasing numbers of patients with complex health needs, and multiple long-term conditions[25]. New models of care in England emerging, for example, through the multispecialty community provider (MCP) contract

For peer review only - http://bmjopen.bmj.com/site/about/guidelines.xhtml

framework[26], need to consider the how to make best use of the primary care workforce. Many GP practices now offer nurse-led consultations for patients with chronic conditions, which are often age-related, such as diabetes. Our results provide some support for this model of care, with approximately one in five patients aged over 65 wanting an appointment with a nurse. Among patients who wanted a nurse appointment and got one, patient-reported trust and confidence in the nurse consultation was very high (mean score 91.0 on a scale of 0 -100). Further work is needed to ascertain the impacts of proposed workforce changes on different patient groups. While proposals for nurses to help meet gaps in the supply of primary care providers have met with wide interest[27], there are important remaining uncertainties about the impact of this on patients' experiences and on the maintenance of confidence and trust in primary care in the NHS.

For peer review only

Last time you wanted to see or speak to a GP or nurse from your GP surgery: What did you want to do?	N (Weighted %)*	What type of appointment did you get?** To see and/or speak to a GP	To see and/or speak to a Nurse	A home visit	One or more of the above	Total
See and/or speak to a GP	653,526 (77.7%)	95.9%	2.9%	0.1%	1.1%	100.0%
See and/or speak to a Nurse	139,300 (14.5%)	6.3%	92.2%	0.1%	1.4%	100.0%
A home visit	12,873 (1.2%)	15.7%	2.2%	79.6%	2.5%	100.0%
Didn't mind/wasn't sure	15,404 (2.4%)	67.1%	29.1%	1.1%	2.7%	100.0%
One or more of the above	48,982 (4.3%)	29.7%	8.6%	0.7%	61.1%	100.0%
Total	870,085 (100.0%)	77.6%	17.5%	1.1%	3.8%	100.0%

*Weighted percentages are calculated using survey design and non-response weights (by age, gender, geographical location and GP practice, full details Technical Annex GP Patient Survey[13])

**Of those who answered the question 'Last time you wanted to see or speak to a GP or nurse from your GP surgery: What did you want to do?'(N = 870,085) only 754,551 also answered the question 'What type of appointment did you get?')

Table 1. Descriptive statistics showing what patients wanted to do last time they contacted their GP surgery, and weighted percentages showing the association between the type of appointment patients wanted and the appointment they got.

For peer review only - http://bmjopen.bmj.com/site/about/guidelines.xhtml

		Wanted GP appointment N (%)†‡	Wanted nurse appointment N (%)†	Crude OR Wanted GP appointment (95% CI)*	Adjusted OR Wanted GP appointment (95% CI)**
Age	18 to 24	23978(86.5%)	3953(13.5%)	1.34 (1.29, 1.39)	1.29 (1.24, 1.34)
	25 to 34	55900(86.1%)	9828(13.9%)	1.26 (1.22, 1.29)	1.20 (1.17, 1.23)
	35 to 44	78731(87.0%)	12575(13.0%)	1.38 (1.35, 1.41)	1.32 (1.29, 1.35)
	45 to 54	106693(85.2%)	19007(14.8%)	1.24 (1.21, 1.27)	1.22 (1.20, 1.24)
	55 to 64	117426(82.3%)	25927(17.7%)	Reference	Reference
	65 to 74	107427(77.9%)	31381(22.1%)	0.76 (0.74, 0.77)	0.77 (0.76, 0.79)
	75 to 84	57635(79.0%)	15802(21.0%)	0.81 (0.79, 0.82)	0.80 (0.79, 0.82)
	85 or over	14857(78.6%)	4124(21.4%)	0.80 (0.77, 0.83)	0.76 (0.74, 0.79)
Gender	Male	246540(85.2%)	53712(14.8%)	Reference	Reference
	Female	316107(82.8%)	68885(17.2%)	1.00 (0.99, 1.01)	0.99 (0.97, 1.00)
Ethnicity	White	495207(83.4%)	113381(16.6%)	Reference	Reference
	Mixed	4432(87.0%)	686(13.0%)	1.48 (1.36, 1.60)	1.23 (1.14, 1.33)
	Asian	33748(89.1%)	4303(10.9%)	1.80 (1.74, 1.85)	1.51 (1.47, 1.56)
	Black	14442(87.8%)	2151(12.2%)	1.54 (1.47, 1.61)	1.30 (1.25, 1.36)
	Other	14818(88.4%)	2076(11.6%)	1.63 (1.56, 1.71)	1.41 (1.35, 1.47)
IMD group	1 – least deprived	111565(83.3%)	25422(16.7%)	Reference	Reference
	2	116118(83.1%)	26848(16.9%)	0.99 (0.97, 1.00)	0.98 (0.97, 1.00)
	3	115657(83.5%)	26334(16.5%)	1.00 (0.98, 1.02)	0.97 (0.96, 0.99)
	4	110059(84.6%)	22543(15.4%)	1.11 (1.09, 1.13)	1.03 (1.01, 1.05)
	5 – most deprived	109248(85.5%)	21450(14.5%)	1.16 (1.14, 1.18)	1.03 (1.01, 1.05)
Confidence in managing health	Very confident	240196(82.3%)	58806(17.7%)	Reference	Reference
	Fairly confident	283776(84.9%)	58435(15.1%)	1.19 (1.17, 1.20)	1.21 (1.19, 1.22)
	Not very confident	33166(89.1%)	4562(10.9%)	1.78 (1.72, 1.84)	1.75 (1.70, 1.80)
	Not at all confident	5509(88.2%)	794(11.8%)	1.70 (1.58, 1.83)	1.73 (1.61, 1.85)
Long term condition	No	216594(85.2%)	42250(14.8%)	Reference	Reference
	Yes	346053(83.0%)	80347(17.0%)	0.84 (0.83, 0.85)	0.97 (0.96, 0.99)

† *Weighted percentages are calculated using survey design and non-response weights (by age, gender, geographical location and GP practice, full details Technical Annex [13])
*p<0.001 for all except gender where p=0.969 **p<0. 001 for all except gender where p = 0. 024 ‡OR prefer nurse come from the same model as prefer GP and are equal to 1/(OR prefer GP)

Table 2. Associations between patient characteristics and wanting an appointment to see or speak to a GP. Odds ratios > 1 indicate patient group more likely to want to see a GP than the reference group. Only those with complete information for all covariates are included (n=728,976)

For peer review only - http://bmjopen.bmj.com/site/about/guidelines.xhtml

		N (%)	Patient-rated confidence and trust	Crude difference* (95% CI)	Adjusted difference** (95% CI)
Consultation outcome: saw/spoke to nurse					
Patient request	Appointment outcome				
See a nurse	Saw a nurse	105713 (87.0%)	91.0	Reference	Reference
See a nurse	Spoke to a nurse	518 (0.4%)	87.6	-3.40 (-5.27, -1.53)	-2.87 (-4.70, -1.04)
Speak to a nurse	Saw a nurse	1165 (1.0%)	87.8	-3.23 (-4.48, -1.98)	-2.82 (-4.05, -1.60)
Speak to a nurse	Spoke to a nurse	1698 (1.4%)	88.3	-2.70 (-3.74, -1.66)	-2.40 (-3.42, -1.38)
See a GP	Saw a nurse	10769 (8.9%)	79.5	-11.58 (-12.01, -11.15)	-9.90 (-10.33, -9.47)
See a GP	Spoke to a nurse	534 (0.4%)	72.9	-18.10 (-19.94, -16.26)	-15.83 (-17.64, -14.02)
Speak to a GP	Saw a nurse	821 (0.7%)	83.9	-7.18 (-8.67, -5.69)	-6.04 (-7.50, -4.58)
Speak to a GP	Spoke to a nurse	286 (0.2%)	76.0	-14.99 (-17.51, -12.48)	-13.47 (-15.93, -11.01)
Consultation outcome: saw/spoke to GP					
Patient request	Appointment outcome				
See a GP	Saw a GP	446631 (90.6%)	84.4	Reference	Reference
See a GP	Spoke to a GP	9000 (1.8%)	76.7	-7.68 (-8.22, -7.13)	-6.47 (-7.00, -5.93)
Speak to a GP	Saw a GP	8190 (1.7%)	83.6	-0.82 (-1.40, -0.25)	-0.77 (-1.33, -0.22)
Speak to a GP	Spoke to a GP	21959 (4.5%)	86.4	1.96 (1.61, 2.32)	1.42 (1.07, 1.76)
See a nurse	Saw a GP	5763 (1.2%)	84.2	-0.19 (-0.87, 0.49)	-0.73 (-1.39, -0.08)
See a nurse	Spoke to a GP	370 (0.1%)	79.2	-5.21 (-7.88, -2.54)	-5.63 (-8.20, -3.07)
Speak to a nurse	Saw a GP	893 (0.2%)	82.9	-1.48 (-3.20, 0.24)	-1.48 (-3.13, 0.18)
Speak to a nurse	Spoke to a GP	173 (0.1%)	80.6	-3.76 (-7.67, 0.14)	-4.04 (-7.80, -0.29)

*p<0.001 (joint test)

**Also adjusted for age, gender, ethnicity, confidence in managing their own health, the presence of a long standing heath condition, deprivation (fixed effects) and practice (random effect) p<0.001 (joint test)

Table 3. Results of the regression analyses examining the association between patient-rated confidence and trust in health professionals, and the difference in the type of appointment patients wanted and the appointment they got at their last consultation in primary care.

For peer review only - http://bmjopen.bmj.com/site/about/guidelines.xhtml

		N (%)	Mean Communication Score	Crude difference* (95% CI)	Adjusted difference** (95% CI)
Consultation outcome: saw/spoke to nurse					
Patient request	Appointment outcome				
See a nurse	Saw a nurse	105140 (86.8%)	90.0	Reference	Reference
See a nurse	Spoke to a nurse	517 (0.4%)	88.3	-1.70 (-2.97, -0.44)	-1.40 (-2.63, -0.18)
Speak to a nurse	Saw a nurse	1170 (1.0%)	87.6	-2.37 (-3.21, -1.53)	-2.05 (-2.87, -1.23)
Speak to a nurse	Spoke to a nurse	1697 (1.4%)	88.9	-1.09 (-1.79, -0.38)	-0.93 (-1.61, -0.24)
See a GP	Saw a nurse	10916 (9.0%)	82.7	-7.31 (-7.60, -7.02)	-5.99 (-6.28, -5.71)
See a GP	Spoke to a nurse	538 (0.4%)	77.8	-12.16 (-13.40, -10.92)	-10.47 (-11.68, -9.27)
Speak to a GP	Saw a nurse	819 (0.7%)	85.7	-4.25 (-5.26, -3.25)	-3.35 (-4.33, -2.38)
Speak to a GP	Spoke to a nurse	289 (0.2%)	80.2	-9.80 (-11.49, -8.11)	-8.66 (-10.30, -7.03)
Consultation outcome: saw/spoke to GP					
Patient request	Appointment outcome				
See a GP	Saw a GP	450555 (90.6%)	85.4	Reference	Reference
See a GP	Spoke to a GP	9127 (1.8%)	80.0	-5.41 (-5.77, -5.05)	-4.49 (-4.84, -4.14)
Speak to a GP	Saw a GP	8281 (1.7%)	85.2	-0.21 (-0.59, 0.17)	-0.23 (-0.59, 0.13)
Speak to a GP	Spoke to a GP	22039 (4.4%)	86.9	1.54 (1.30, 1.77)	0.96 (0.73, 1.19)
See a nurse	Saw a GP	5831 (1.2%)	84.5	-0.85 (-1.30, -0.40)	-1.22 (-1.65, -0.80)
See a nurse	Spoke to a GP	378 (0.1%)	82.0	-3.41 (-5.17, -1.64)	-3.76 (-5.43, -2.10)
Speak to a nurse	Saw a GP	913 (0.2%)	84.3	-1.07 (-2.21, 0.07)	-0.94 (-2.01, 0.13)
Speak to a nurse	Spoke to a GP	178 (0.0%)	83.7	-1.64 (-4.21, 0.93)	-2.16 (-4.59, 0.26)

*p<0.001 (joint test)

**Also adjusted for age, gender, ethnicity, confidence in managing their own health, the presence of a long standing heath condition, deprivation (fixed effects) and practice (random effect) p<0.001 (joint test)

Table 4 – Results of regression analyses examining the association between patient rated nurse and GP communication scores, and the difference in the type of appointment patients wanted and the appointment they got at their last consultation in primary care.

For peer review only - http://bmjopen.bmj.com/site/about/guidelines.xhtml

For peer review only

For peer review only - http://bmjopen.bmj.com/site/about/guidelines.xhtml

Checklists

A STROBE checklist is included as a supplementary file.

Exclusive license

The Corresponding Author has the right to grant on behalf of all authors and does grant on behalf of all authors, a worldwide license to the Publishers and its licensees in perpetuity, in all forms, formats and media (whether known now or created in the future), to i) publish, reproduce, distribute, display and store the Contribution, ii) translate the Contribution into other languages, create adaptations, reprints, include within collections and create summaries, extracts and/or, abstracts of the Contribution, iii) create any other derivative work(s) based on the Contribution, iv) to exploit all subsidiary rights in the Contribution, v) the inclusion of electronic links from the Contribution to third party material where-ever it may be located; and, vi) license any third party to do any or all of the above.

Contributorship statement

All authors (CP, GA, JB, JC, ME, VL, and MR) jointly conceived of the research questions, reviewed/edited the manuscript and contributed to discussion and interpretation of the data. CP and GA led on writing the manuscript, and GA completed the statistical analysis. All authors (CP, GA, JB, JC, ME, VL, and MR) had full access to all of the data in the study and take responsibility for the integrity of the data and accuracy of the data analysis. CP is the guarantor of the paper.

Ethical approval

The study did not require ethical approval as it is an observational study based on secondary analysis of data from a national survey (the English General Practice Patient Survey) conducted and funded by NHS England.

Competing interests

We have read and understood BMJ Open policy on declaration of interests and declare that we have no competing interests.

Data sharing

No additional data are available.

Transparency declaration.

Dr Paddison affirms that the manuscript is an honest, accurate, and transparent account of the study being reported; that no important aspects of the study have been omitted. There are no significant discrepancies from the study as planned.

Funding

This paper presents independent research funded by the National Institute for Health Research (NIHR) under its Programme Grants for Applied Research Programme (Grant Reference Number RP-PG-0608-10050). The views expressed are those of the author(s) and not necessarily those of the

For peer review only - http://bmjopen.bmj.com/site/about/guidelines.xhtml

NHS, the NIHR or the Department of Health. The researchers confirm their independence from the study funders, the National Institute for Health Research.

Open Access statement

This is an open access article distributed in accordance with the terms of the Creative Commons Attribution (CC BY 4.0) license, which permits others to distribute, remix, adapt and build upon this work, for commercial use, provided the original work is properly cited. See: http://creativecommons.org/licenses/by/4.0

Sponsorship

The study was sponsored by the University of Cambridge.

For peer review only

REFERENCES

1 Campbell SM, Roland MO, Buetow SA. Defining quality of care. *Soc Sci Med* 2000;**51**:1611–25.http://www.sciencedirect.com/science/article/pii/S0277953600000575

2 Department of Health. The NHS Outcomes Framework 2011/12. 2010. http://www.dh.gov.uk/en/Publicationsandstatistics/Publications/PublicationsPolicyAndGuidance/DH_122944

3 Department of Health. Equity and Excellence: Liberating the NHS. 2010. https://www.gov.uk/government/publications/liberating-the-nhs-white-paper

4 House of Commons Health Committee. Primary Care: Fourth Report of Session 2015–16. 2016. http://www.publications.parliament.uk/pa/cm201516/cmselect/cmhealth/408/408.pdf.

5 Roland M, Everington S. Tackling the crisis in general practice. *BMJ* 2016;**352**:i942. doi:10.1136/bmj.i942

6 NHS England. General Practice Forward View. 2016. https://www.england.nhs.uk/wp-content/uploads/2016/04/gpfv.pdf

7 Primary Care Workforce Commission. The future of primary care: Creating teams for tomorrow. 2015.https://hee.nhs.uk/sites/default/files/documents/The%20Future%20of%20Primary%20Care%20report.pdf

8 Horrocks S, Anderson E, Salisbury C. Systematic review of whether nurse practitioners working in primary care can provide equivalent care to doctors. *BMJ* 2002;**324**:819–23. doi:10.1136/bmj.324.7341.819

9 Laurant M, Reeves D, Hermens R, *et al.* Substitution of doctors by nurses in primary care. *Cochrane Database Syst Rev* Published Online First: 2005. doi:10.1002/14651858.CD001271.pub2

10 Hegney D, Price K, Patterson E, *et al.* Australia consumers' expectations for expanded nursing roles in general practice - choice not gatekeeping. *Aust Fam Physician* 2004;**33**:845–9.

11 Redsell S, Stokes T, Jackson C, *et al.* Patients' accounts of the differences in nurses' and general practitioners' roles in primary care. *J Adv Nurs* 2007;**57**:172–80. doi:10.1111/j.1365-2648.2006.04085.x

12 Campbell J, Smith P, Nissen S, *et al.* The GP Patient Survey for use in primary care in the National Health Service in the UK - development and psychometric characteristics. *BMC Fam Pract* 2009;**10**:57.http://www.biomedcentral.com/1471-2296/10/57

13 Ipsos MORI. GP Patient Survey - Technical annex. 2014.

14 Lyratzopoulos G, Elliott M, Barbiere JM, *et al.* Understanding ethnic and other socio-demographic differences in patient experience of primary care: evidence from the English General Practice Patient Survey. *BMJ Qual Saf* 2012;**21**:21–9. doi:10.1136/bmjqs-2011-000088

15 Roberts MJ, Campbell JL, Abel GA, *et al.* Understanding high and low patient experience scores in primary care: analysis of patients' survey data for general practices and individual doctors. *BMJ* 2014;**349**:g6034. doi:10.1136/bmj.g6034

16 Halcomb EJ, Peters K, Davies D. A qualitative evaluation of New Zealand consumers perceptions of general practice nurses. *BMC Fam Pract* 2013;**14**:26. doi:10.1186/1471-2296-14-26

17 Parker R, Forrest L, Ward N, *et al.* How acceptable are primary health care nurse practitioners to Australian consumers? *Coll R Coll Nurs Aust* 2013;**20**:35–41.

18 Martínez-González NA, Djalali S, Tandjung R, *et al.* Substitution of physicians by nurses in primary care: a systematic review and meta-analysis. *BMC Health Serv Res* 2014;**14**:214. doi:10.1186/1472-6963-14-214

19 Rashidian A, Shakibazadeh E, Karimi- Shahanjarini A, *et al.* Barriers and facilitators to the implementation of doctor-nurse substitution strategies in primary care: qualitative evidence synthesis. In: *Cochrane Database of Systematic Reviews*. 2013. doi:10.1002/14651858.CD010412

20 Lyratzopoulos G, Barbiere J, Elliott M, *et al.* How can healthcare providers be reliably compared? Lessons from a national survey of patient experience. *Med Care* 2011;**49**:724–33.

21 Campbell JL, Fletcher E, Britten N, *et al.* Telephone triage for management of same-day consultation requests in general practice (the ESTEEM trial): a cluster-randomised controlled trial and cost-consequence analysis. *The Lancet* 2014;**384**:1859–68. doi:10.1016/S0140-6736(14)61058-8

22 Halter M, Drennan VM, Joly LM, Gabe J, *et al.* Patients' experiences of consultations with physician associates in primary care in England: A qualitative study. *Health Expect.* 2017;20:1011–1019. https://doi.org/10.1111/hex.12542

23 Healey EL, Main CJ, Ryan S, *et al.* A nurse-led clinic for patients consulting with osteoarthritis in general practice: development and impact of training in a cluster randomised controlled trial. *BMC Family Practice*. 2016;17:173. doi:10.1186/s12875-016-0568-y.

24 Varley A, Warren FC, Richards SH, *et al*. The effect of nurses' preparedness and nurse practitioner status on triage call management in primary care: A secondary analysis of cross-sectional data from the ESTEEM trial. *International Journal of Nursing Studies*. 2016;58:12-20. doi:10.1016/j.ijnurstu.2016.02.001.

For peer review only - http://bmjopen.bmj.com/site/about/guidelines.xhtml

25 Barnett K, Mercer SW, Norbury M, *et al.* Epidemiology of multmorbidity and implications for health care, research, and medical education: a cross-sectional study. *The Lancet* 2012;**380**:37–43.

26 NHS England. Multispeciality community (MCP) emerging care model and contract framework. 2016.https://www.england.nhs.uk/wp-content/uploads/2016/07/mcp-care-model-frmwrk.pdf

27 Donelan K, DesRoches CM, Dittus RS, *et al.* Perspectives of Physicians and Nurse Practitioners on Primary Care Practice. *N Engl J Med* 2013;**368**:1898–906. doi:10.1056/NEJMsa1212938

For peer review only

For peer review only - http://bmjopen.bmj.com/site/about/guidelines.xhtml

Appendix Table 1. Five category summary of responses to the question “What did you want to do?” in the GP Patient Survey

	Response options to question: Last time you wanted to see or speak to a GP or nurse from your GP surgery: What did you want to do?*					
	See a GP at the surgery	See a nurse at the surgery	Speak to a GP on the phone	Speak to a nurse on the phone	Have someone visit me at my home	I didn’t mind / wasn’t sure what I wanted
Wanted to see or speak to a GP (or both)	**X**					
			**X**			
	**X**		**X**			
Wanted to see or speak to a Nurse (or both)		**X**				
				**X**		
		**X**		**X**		
Wanted a home visit					**X**	
Wasn’t sure or didn’t mind						**X**
Wanted more than one of the previous four categories	Any Other combination					

*Response options available to primary care patients completing the 2013/14 General Practice Patient Survey (http://www.gp-patient.co.uk).

For peer review only

Appendix Table 2. Two category summary of responses to the question “What did you want to do?” in the GP Patient Survey

	Last time you wanted to see or speak to a GP or nurse from your GP surgery: What did you want to do?					
	See a GP at the surgery	See a nurse at the surgery	Speak to a GP on the phone	Speak to a nurse on the phone	Have someone visit me at my home	I didn’t mind / wasn’t sure what I wanted
Wanted to see or speak to a GP (or both)	X					
			X			
	X		X			
	X					X*
			X			X*
	X		X			X*
Wanted to see or speak to a Nurse (or both)		X				
				X		
		X		X		
		X				X*
				X		X*
		X		X		X*
Excluded	Any other combination					

*Where a patient indicated they didn’t mind while only indicating an appointment with either a GP or nurse, but not both, we took this to indicate they did not mind how they interacted with the healthcare professional, but they had expressed a preference for a particular type of health professional

For peer review only

STROBE Statement—checklist of items that should be included in reports of observational studies

	Item No.	Recommendation	Page No.	Relevant text from manuscript
**Title and abstract**	1	(*a*) Indicate the study's design with a commonly used term in the title or the abstract	1	Study type is in the title and abstract
		(*b*) Provide in the abstract an informative and balanced summary of what was done and what was found	1	Abstract
		**Introduction**		
Background/rationale	2	Explain the scientific background and rationale for the investigation being reported	2	Background section
Objectives	3	State specific objectives, including any pre-specified hypotheses	2	Two objectives are specified.
		**Methods**		
Study design	4	Present key elements of study design early in the paper	2-3	Study design is in both the background and the method section.
Setting	5	Describe the setting, locations, and relevant dates, including periods of recruitment, exposure, follow-up, and data collection	2-3	Method section
Participants	6	(*a*) *Cohort study*—Give the eligibility criteria, and the sources and methods of selection of participants. Describe methods of follow-up		N/A
		Case-control study—Give the eligibility criteria, and the sources and methods of case ascertainment and control selection. Give the rationale for the choice of cases and controls		N/A
		Cross-sectional study—Give the eligibility criteria, and the sources and methods of selection of participants	2-3	Selection of patients to include described in method section.
		(*b*) *Cohort study*—For matched studies, give matching criteria and number of exposed and unexposed		N/A
		Case-control study—For matched studies, give matching criteria and the number of controls per case		N/A
Variables	7	Clearly define all outcomes, exposures, predictors, potential confounders, and effect modifiers. Give diagnostic criteria, if applicable	2-3	Outcome s are specified questions in the GP Patient Survey

For peer review only - http://bmjopen.bmj.com/site/about/guidelines.xhtml

Data sources/ measurement	8*	For each variable of interest, give sources of data and details of methods of assessment (measurement). Describe comparability of assessment methods if there is more than one group	2,5	We report on the response rate to the survey and published evidence that this is unlikely to be associated with major biases in the data
Bias	9	Describe any efforts to address potential sources of bias	2-3	We adjusted for major potential confounders. Other details are referenced to our previous approach to analysing these data (references 14 and 15) and to the IPSOS MORI Technical Annex to the GP Patient Survey (reference 13)
Study size	10	Explain how the study size was arrived at	2-3	We used all data that met our eligibility criteria from respondents to one year of the GP Patient Survey (870.085 respondents). There was no formal sample size calculation: with these numbers, statistical significance is likely to be reached for very small effects. Of greater relevance is the interpretation of small but statistically significant differences.

Continued on next page

For peer review only - http://bmjopen.bmj.com/site/about/guidelines.xhtml

Quantitative variables	11	Explain how quantitative variables were handled in the analyses. If applicable, describe which groupings were chosen and why	2-3	See responses to 6,7 and 9 above.
Statistical methods	12	(*a*) Describe all statistical methods, including those used to control for confounding	2-3	Potential confounders are listed in the method section and included as adjusted estimates of effect size (which are the main ones we report).
		(*b*) Describe any methods used to examine subgroups and interactions		As above
		(*c*) Explain how missing data were addressed		See answer to question 9. These references (14 and 15) also describe how we dealt with patients who did not complete all items in the communication section of the GP Patient Survey.
		(*d*) *Cohort study*—If applicable, explain how loss to follow-up was addressed		N/A
		Case-control study—If applicable, explain how matching of cases and controls was addressed		N/A
		Cross-sectional study—If applicable, describe analytical methods taking account of sampling strategy	2-3	We describe how we used survey weights to account for survey design and non-response.
		(*e*) Describe any sensitivity analyses		None
**Results**				
Participants	13*	(a) Report numbers of individuals at each stage of study—eg numbers potentially eligible, examined for eligibility, confirmed eligible, included in the study, completing follow-up, and analysed	4	Results section
		(b) Give reasons for non-participation at each stage		N/A
		(c) Consider use of a flow diagram		Not required
Descriptive data	14*	(a) Give characteristics of study participants (eg demographic, clinical, social) and information on exposures and potential confounders	8	Listed in table 2 – these were included in the adjusted estimates in tables 3 and 4.
		(b) Indicate number of participants with missing data for each variable of interest	4	We used only subjects with complete data (n=685,244)
		(c) *Cohort study*—Summarise follow-up time (eg, average and total amount)		N/A

For peer review only - http://bmjopen.bmj.com/site/about/guidelines.xhtml

Outcome data	15*	*Cohort study*—Report numbers of outcome events or summary measures over time		N/A
		Case-control study—Report numbers in each exposure category, or summary measures of exposure		N/A
		Cross-sectional study—Report numbers of outcome events or summary measures	2-4	Two main outcomes as described in the introduction (objectives) and method sections.
Main results	16	(*a*) Give unadjusted estimates and, if applicable, confounder-adjusted estimates and their precision (eg, 95% confidence interval). Make clear which confounders were adjusted for and why they were included	9-10	Tables 3 and 4 give both unadjusted (crude) and adjusted estimates.
		(*b*) Report category boundaries when continuous variables were categorized		N/A
		(*c*) If relevant, consider translating estimates of relative risk into absolute risk for a meaningful time period		N/A

Continued on next page

For peer review only

Other analyses	17	Report other analyses done—eg analyses of subgroups and interactions, and sensitivity analyses		No other analyses
**Discussion**				
Key results	18	Summarise key results with reference to study objectives	5	Discussion
Limitations	19	Discuss limitations of the study, taking into account sources of potential bias or imprecision. Discuss both direction and magnitude of any potential bias	5	Discussion
Interpretation	20	Give a cautious overall interpretation of results considering objectives, limitations, multiplicity of analyses, results from similar studies, and other relevant evidence	5-6	Discussion
Generalisability	21	Discuss the generalisability (external validity) of the study results	5	Discussed in terms of this being a strength of the study, as it uses a large national survey with data from over 8000 general practices (i.e. almost all those in England)
**Other information**				
Funding	22	Give the source of funding and the role of the funders for the present study and, if applicable, for the original study on which the present article is based	14	The study was funded by the National Institute for Health Research (programme grant RP-PG-0608-10050)

*Give information separately for cases and controls in case-control studies and, if applicable, for exposed and unexposed groups in cohort and cross-sectional studies.

Note: An Explanation and Elaboration article discusses each checklist item and gives methodological background and published examples of transparent reporting. The STROBE checklist is best used in conjunction with this article (freely available on the Web sites of PLoS Medicine at http://www.plosmedicine.org/, Annals of Internal Medicine at http://www.annals.org/, and Epidemiology at http://www.epidem.com/). Information on the STROBE Initiative is available at www.strobe-statement.org.

For peer review only

For peer review only - http://bmjopen.bmj.com/site/about/guidelines.xhtml
